# Supplementary material for: Retrospective Detection and Genetic Characterization of Porcine circovirus 3 (PCV3) Strains Identified between 2006 and 2007 in Brazil
Source: Viruses. 2019 Feb 27;11(3):201. doi: 10.3390/v11030201 (PMC6466443; doi:10.3390/v11030201)
Supplement: Supplementary file 1 [file viruses-11-00201-s001.pdf]

Supplementary Material

Table 1. Detailed information of 67 tissue samples collected from pigs between 2006 and 2007 from nine states of Brazil.

Strain	GenBank Accession	Geographical origin	Year of collection	Clinical signs	PCV2 infection	PCV3 infection
68		Nd #	2007	-	-	+
70		Minas Gerais	2007	-	-	+
78		Minas Gerais	2006	-	-	-
137		Minas Gerais	2007	-	-	+
147		São Paulo	2006	-	-	-
200		Minas Gerais	2006	-	+	+
295*	MK060075	Espírito Santo	2007	+	+	+
297		Minas Gerais	2007	-	-	+
348		Minas Gerais	2007	-	-	-
408		Minas Gerais	2007	+	+	-
474		Minas Gerais	2006	-	-	-
488		São Paulo	2007	+	+	+
489		Minas Gerais	2007	+	+	-
526		Espírito Santo	2007	+	+	+
532		Minas Gerais	2007	-	-	+
600		Mato Grosso	2007	+	+	+
622*	MK060079	Mato Grosso	2007	-	-	+
633		Minas Gerais	2007	-	-	-
648		Minas Gerais	2007	-	-	+
666		Rio Grande do Sul	2007	+	+	+
679		Minas Gerais	2007	-	-	-
727		Minas Gerais	2007	+	+	+
837		Mato Grosso	2007	+	+	+
839		Paraná	2007	-	-	+
872*	MK060076	Goiás	2007	-	-	+
876		Minas Gerais	2007	-	-	+
893		Minas Gerais	2007	-	-	-
916*	MK060074	Paraná	2007	-	-	+
955		Minas Gerais	2006	-	-	-
958		Mato Grosso	2007	-	-	-
966		Mato Grosso	2007	-	-	+
986*	MK060078	Mato Grosso do Sul	2007	+	+	+
987		Minas Gerais	2006	-	-	-
988		Minas Gerais	2007	-	-	+
993		Minas Gerais	2007	-	-	+
1029		Minas Gerais	2007	-	-	+
1034		Goiás	2007	+	+	-
1042		Minas Gerais	2007	-	-	-
1050		Minas Gerais	2007	-	-	+

1061		Minas Gerais	2007	-	-	-
1066		Minas Gerais	2006	-	-	-
1107		Minas Gerais	2006	-	-	-
1125		Minas Gerais	2007	+	+	+
1144		Santa Catarina	2007	+	+	-
1155		Minas Gerais	2007	-	-	-
1162		Minas Gerais	2007	+	+	-
1163		Minas Gerais	2007	+	+	-
1164		Minas Gerais	2007	-	-	+
1167		Minas Gerais	2006	-	+	-
1169		Minas Gerais	2007	-	-	-
1171		Minas Gerais	2007	+	+	-
1218		Minas Gerais	2007	+	+	-
1222		Minas Gerais	2007	+	+	-
1231		Minas Gerais	2007	+	+	-
1291		Minas Gerais	2007	-	-	-
1322		Minas Gerais	2006	+	+	-
1329		Rio Grande do Sul	2007	-	-	+
1343*	MK060073	Minas Gerais	2006	+	+	+
1350		Minas Gerais	2007	-	-	-
1373*	MK060077	São Paulo	2007	-	-	+
1396		Rio Grande do Sul	2007	-	-	+
1405		Minas Gerais	2006	+	+	-
1410		Minas Gerais	2006	+	+	-
1429		Santa Catarina	2007	+	+	+
1445		Nd #	2006	-	-	-
1456		Nd #	2007	-	-	-
1473		Minas Gerais	2006	+	+	-

*Sequenced samples # Nd= no data available
